# Development and Characteristics of Sexting from Age 14 to 18 Years in a Norwegian Birth Cohort

**DOI:** 10.1007/s10508-026-03413-5

**Published:** 2026-03-17

**Authors:** Silje Steinsbekk, Jacqueline Nesi, Sophia Choukas-Bradley, Lars Wichstrøm

**Affiliations:** 1 Department of Psychology, Norwegian University of Science and Technology, Dragvoll, 7491 Trondheim, Norway; 2 Department of Psychiatry & Human Behavior, Warren Alpert Medical School of Brown University, Providence, RI USA; 3 Department of Psychology, University of Pittsburgh, Pittsburgh, PA USA; 4 Department of Child and Adolescent Psychiatry, St. Olavs University Hospital, Trondheim, Norway

**Keywords:** Sexting, Sending, Receiving, Adolescents, Social media, Trondheim Early Secure Study

## Abstract

Sexting, the sending or receiving of sexually explicit texts, images, or videos via technological devices, is common among adolescents. This study examined sexting behaviors in a birth cohort assessed at ages 14, 16, and 18 years (analytical sample: n = 743, 53.7% girls). Results showed receiving sexts was more common than sending them, with frequency increasing with age. Both sending and receiving sexts exhibited significant two-year rank-order stability. Sexting typically occurred on non-anonymous social media platforms (e.g., Snapchat, Instagram), with fewer than 5% using anonymous platforms. Adolescents mostly sent sexts to boyfriends or girlfriends, with the proportion rising from 19% to 27% between ages 16 and 18. At age 14, 19% reported receiving sexts from unknown people, doubling by age 18. The prevalence of sending sexts did not significantly differ between girls and boys, but girls were more likely to receive sexts, especially from unknown individuals and on their own social media platforms. Sex, pubertal status, and biological age did not predict changes in sexting frequency from age 14 to 18. The study highlights the need for interventions and policies that recognize certain types of sexting as part of sexual development while protecting against sexual victimization and associated risks.

**Supplementary Information:**

The online version contains supplementary material available at 10.1007/s10508-026-03413-5.

## Introduction

Sexting, defined as sending or receiving sexually explicit messages, images or videos using technological devices (e.g., mobile phones, computers), is relatively common among adolescents worldwide. According to meta-analyses, 15–19% of adolescents report having sent, while 27–35% have received sexts (Madigan et al., 2018; Mori et al., 2022). From a developmental perspective, sexting can be seen as part of natural sexual exploration during adolescence and may contribute to relational and body image development (Bianchi et al., 2017; Lebedíková et al., 2024; Mori et al., 2022; Temple et al., 2019; Thorne et al., 2024; Widman et al., 2021). At the same time, sexting has also been associated with risky behaviors and mental health problems, though findings remain mixed (Doyle et al., 2021; Gassó et al., 2019; Hu et al., 2023; Mariamo et al., 2024; Mori et al., 2019). As a relatively new behavior, many questions remain about the characteristics and developmental course of sexting during adolescence. This study aimed to fill this gap by examining sexting behaviors using three waves of biennially collected data. Specifically, we extend prior research by studying sexting behaviors within a birth-cohort rather than in convenience or school samples and exploring a longer age period. Our study investigated the trajectory of sexting (i.e., individual development, mean level changes, stability) and the characteristics of senders and receivers, as well as the platforms used for sending and receiving image- and video-based sexts.

A recent review identified 24 longitudinal studies of sexting, with the majority conducted in the U.S. and including adolescent samples. Most studies captured two annual waves only (Hu et al., 2023), and thus were not well positioned to capture developmental trends. Seven studies relied on 3 or 4 follow-ups, capturing a 4-year period at the most (Hu et al., 2023), and only four examined how sexting evolved over time (Choi et al., 2019; Hicks et al., 2021; Steinberg et al., 2019; Van Ouytsel et al., 2019). The above studies found that sexting increased by age (Choi et al., 2019) and that sexting at age 16 predicted future sexting (Hicks et al., 2021; Van Ouytsel et al., 2019). Given that sexting is indeed evident in adolescents younger than age 16 (e.g., Burén & Lunde, 2018), longitudinal studies starting at an earlier age are needed. Further, a meta-analysis reported a higher prevalence of sending sexts in older compared to younger adolescents, whereas no age-differences were found for receiving sexts (Mori et al., 2022), thus suggesting that different developmental trajectories may exist for sending and receiving. The above longitudinal studies did not differentiate between these sexting behaviors; thus, it is yet to be revealed whether such developmental differences exist. It should also be noted that although findings are mixed (Barrense-Dias et al., 2017; Barroso et al., 2023), one meta-analysis found that female adolescents were more likely than male adolescents to receive sexts, whereas no differences emerged for sending. (Mori et al., 2022). Failing to differentiate between sending and receiving may thus conceal potential sex differences. According to biosocial role theory (originally social role theory) (Eagly & Wood, 1999), differing societal expectations of gender roles lead men and women to display different sexual behaviors. Developmental theories, such as the bioecological model (Bronfenbrenner, 1979; Bronfenbrenner & Morris, 2007) stress that social and cultural factors affect adolescents’ behavior and thus likely influence their sexting behavior. Based on these frameworks, one would expect sex differences in sexting to be less evident in more sexually liberal and gender-equal societies. However, Mori et al.’s meta-analysis—covering studies from Europe and the US—did not find geographical location to moderate sex differences in sexting rates. Similarly, studies comparing Spanish adolescents with their Mexican and Colombian counterparts also did not find such moderation (Gil-Llario et al., 2020, 2021). The present study contributes to existing research by examining sex differences in one of the most gender-equal and sexually liberal countries in the world. It not only captures differences in rates of sending and receiving sexts but also explores whether sex differences exist in terms of whom adolescents send sexts to and receive sexts from, as well as which platforms they use.

Furthermore, sexting is associated with pubertal status (Burén & Lunde, 2018; Houck et al., 2014), likely due to more physically mature individuals being more interested in and more likely to have sex. Given the risks that come with sexting (e.g., grooming) we need to determine whether “early bloomers” are more prone to sexting and, consequently, more vulnerable to its potential negative consequences. As pubertal status is highly correlated with age and biological sex (e.g., girls are about 2 years ahead of boys [Emmanuel & Bokor, 2023]), it is essential to adjust for these factors to isolate the potential impact of puberty. We therefore studied the role of puberty, age and biological sex, adjusted for each other, in predicting receiving and sending sexts.

The potential negative outcomes of sexting may differ according to whom the sender and receiver are, and whether anonymous (e.g., Jodel) or non-anonymous (e.g., Instagram) platforms are used. According to Valkenburg and Peter (2011), “source anonymity” (i.e., when the individual participating in the online communication cannot be identified (Qian & Scott, 2007)) affects the individual’s self-presentation and self-disclosure, potentially causing less concern about others’ evaluation of oneself, but also increasing the likelihood of impulsive online behavior. In the context of sexting, such anonymity may therefore give adolescents an opportunity to use sexting—as part of a natural and age-appropriate sexual exploration—in a more free or non-shameful way. On the other hand, the “deindividuation” (Joinson, 2001) characterizing anonymous online communication may cause adolescents to be more lenient in sending sexts, thus increasing the overall frequency and perhaps making it more likely that they impulsively send sexts they later regret. This might put them at increased risk for the potential negative outcomes of sexting, such as perpetration, sexual victimization, and feelings of shame or humiliation (Doyle et al., 2021). Whether youth receive sexually explicit messages or images from individuals they do versus do not know may also differently affect them. Indeed, the characteristics and risk profile of sexting has been found to differ depending on the context of the relationship in which it occurs. For example, sexting has been associated with risky behaviors such as substance abuse, but not if it takes place within a committed relationship (Van Ouytsel et al., 2018). Further, if the sender of a sext is unknown, it might be experienced as more intrusive, and thus have more negative impact, compared to when the sender is known. In the context of non-consensual sexts on anonymous forums, victims are involuntarily exposed to unsolicited sexual material, which in some cases may be perceived as an act of aggression and violence (Barroso et al., 2023). We therefore aimed to identify the nature of the sender and receiver of sexts, as well as the anonymity of the platforms used, at different ages in the current study.

In sum, we aimed to characterize the developmental course and characteristics of sexting by addressing the following research questions: (1) What is the lifetime prevalence of sending and receiving sexts (i.e., images and videos) at ages 14, 16, and 18 in a Norwegian birth cohort? (2) To whom do adolescents send and from whom do they receive sexts? (3) Which online platforms do they use for sexting? (4) Are there age and sex differences in whom they send sexts to, whom they receive sexts from, and which platforms they use to engage in sexting? (5) Are the two sexting behaviors—sending and receiving—associated? (6) To what extent is the frequency of sending and receiving sexts stable (i.e., relative to the group) over time? (7) How does the mean frequency of sending and receiving sexts evolve from age 14 to 18? (8) Are individual differences in this development of sexting predicted by sex, age, and pubertal status?

## Method

### Participants and Procedure

The present study utilized data from the Trondheim Early Secure Study (TESS), a longitudinal study of children’s mental health, psychosocial development and health behavior (Steinsbekk & Wichstrøm, 2018). In 2007/2008, all children born in 2003 and 2004 in Trondheim, Norway (N = 3,456) were invited to participate. Parents received an invitation letter along with the Strengths and Difficulties Questionnaire (SDQ) version 4–16 (Goodman, 1997), which they brought to their child’s 4-year health check at the community health center. During the check-up, healthcare nurses informed parents about the study and obtained written consent. Nearly all children (97.2%) attended the check-up, and 82.1% of those invited consented to participate.

To increase statistical power, children with higher SDQ scores were oversampled. Participants were divided into four strata based on their SDQ scores (cut-offs: 0–4, 5–8, 9–11, and 12–40), with selection probabilities increasing with higher scores (.37, .48, .70, and .89, respectively). This procedure resulted in the selection of 1,250 participants, of whom 1007 (79.8%) attended the first assessment at the university clinic. Assessments have been conducted at the university clinic every two years since then, with eight waves of data in total (Figure S1). Sexting behavior was assessed from age 14 onwards, thus the present study uses data collected at ages 14 (2017–2019, n = 635, 52.9% girls, 47.1% boys, Mage = 14.35, SD = .16), 16 (2020–2022, n = 664, 55.0% girls, 45.0% boys, Mage = 16.98, SD = .31), and 18 (2022–2023, n = 664, 54.5% girls, 45.4% boys, Mage = 18.6, SD = .24). At each measurement point, participants completed the questionnaire privately. Participants with information from at least one data wave composed the analytical sample (n = 743). Attrition analyses showed that neither the frequency of sending nor receiving sexts predicted dropout at any time point, but attrition was higher among boys compared to girls (age 14–16: OR = 1.76, 95% CI [1.37, 2.25], *p* ≤ .001; age 16–18: OR = 1.64, 95% CI [1.28, 2.09], *p* ≤ .001). Pubertal status was also not significantly related to dropout.

The TESS sample is representative of the Norwegian population in terms of parents’ education levels and family situation (e.g., proportion of parents being married/cohabitants) (Statistics Norway, 2012, 2017). At baseline, 88.9% of biological parents were married or cohabiting, 92.8% had completed senior high school or higher education, and the majority were ethnic Norwegians (mothers: 90.4%; fathers: 88.2%).

### Measures

At all three measurement points (ages 14, 16, and 18), participants responded to 8 items capturing whether they had ever (1) sent to others or published online an image or video of themselves wearing little clothing (e.g., with breasts or genitals partly visible), and (2) received an image or video of another person who was wearing little clothing. The items also assessed to whom such content had been sent, from whom it had been received, and which platforms were used (e.g., their own social media accounts, anonymous platforms, or other platforms). All items and response options included in the questionnaire are displayed in Table 1.

**Table 1:** Sexting questions at ages 14, 16, and 18 years

Questions		Response options
1	Have you ever sent to others or published online a picture or video of yourself where you were wearing little clothing (e.g., where breasts or genitals are somewhat visible)? (If no, go to q. 5)	1 = Yes 2 = No
2	If yes: How many times have you sent/published such pictures/videos?	1 = Once 2 = 2—3 times 3 = 4—6 times 4 = 7 times or more
3	If yes: To whom have you sent such pictures/videos? Check one or more boxes	1 = People you don’t know (e.g., posted on web platforms, sent anonymous messages) 2 = Friends 3 = Boyfriend/girlfriend 4 = Others
4	If yes: Where have you sent such pictures/videos? Check one or more boxes	1 = Anonymous platforms 2 = Social media platforms (e.g., Facebook, Snap, Instagram, not anonymously) 3 = Other places
5	Have you ever received* a picture or video of someone else where they were wearing little clothing (e.g., where breasts or genitals were somewhat visible)?	1 = Yes 2 = No
6	If yes: How many times have you received such pictures/videos?	1 = Once 2 = 2–3 times 3 = 4–6 times 4 = 7 times or more
7	If yes: From whom have you received such pictures/videos? Check one or more boxes	1 = People you don’t know (e.g., posted on web platforms, sent anonymous messages) 2 = Friends 3 = Boyfriend/girlfriend 4 = Others
8	If yes: Where were these pictures/videos published? Check one or more boxes	1 = Anonymous platforms 2 = Social media platforms (e.g., Facebook, Snap, Instagram, not anonymously) 3 = Other places

*Note* *The Norwegian expression “mottatt” (received) would most likely be interpreted as something sent intentionally, although not necessarily personally

Sex was coded based on the participant’s national ID number, which is based on sex assigned at birth. Pubertal status was assessed using the Pubertal Development Scale (PDS) (Petersen et al., 1988). For each sex, a mean score was calculated based on participants’ responses on 5 questions assessing different aspects of physical development (e.g., boys: facial hair; girls: breast development; both sexes: growth spurt), with response options on a 4-point Likert scale, with higher scores reflecting further progress in development (e.g., for breast development, the scale ranged from “not started yet” to “it seems like my breasts are fully developed”). Based on mean scores, participants were grouped into the following 5 categories according to Tanner’s criteria: “Pre-Pubertal”; “Beginning-Pubertal”; “Mid-pubertal”; “Advanced-Pubertal”; “Post-Pubertal.”

### Statistical Analyses

Estimation of the lifetime prevalence of sexting behavior was performed in SPSS version 29.0 using population weights to account for the initial oversampling of children with higher SDQ scores and applying a sandwich estimator to provide accurate confidence intervals. All remaining analyses were conducted in Mplus 8.11 (Muthén & Muthén, 1998–2017), using a robust maximum likelihood (MLR) estimator and applying population weights. To examine age differences in prevalence and characteristics of sexting, we estimated a model where the thresholds of the categorical variable in question (e.g., sending nudes to boyfriend/girlfriend at ages 14 and 16) were freely estimated and compared it with a model where the thresholds were constrained to be equal across age by means of a chi-square difference test using the log-likelihood (Muthen & Muthen, 2025; Satorra & Bentler, 2001, 2010). If this test shows that the fit deteriorates when age constraints are added to the model, it indicates that age differences do exist (i.e., the thresholds cannot be constrained to be equal at the two time points). To examine potential gender differences, we conducted logistic regression analyses, one for each variable at each age (e.g., receiving nudes from boyfriend/girlfriend at age 16), regressing the variable in question on sex. Bivariate correlations were estimated to examine the cross-sectional associations between sending and receiving and to test stability relative to the group across age in each of these sexting behaviors.

To examine development in sexting from age 14 to 18 years, we fitted a latent growth model estimating the intercept and the growth of the frequency of sending and receiving sexts, respectively. As displayed in Table 1, four categories of frequency of sending and receiving sexts were included, with some of these categories capturing a range of frequencies (e.g., response option 3 = 4–6 times). To estimate more readily interpretable means and growth estimates, we therefore applied recoded frequency variables in the growth analyses (0 = 0; 1 = 1; 2 = 2.5 (originally 2–3); 3 = 5 (originally 4–6); 7 = 10 (originally 7 times or more)). Please note that 0 was not a response option for the frequency variable but was coded based on the response “No” on the initial question (i.e., have you ever sent/received?).

The intercept of the growth model represents the starting point of the growth, and growth was parameterized as yearly change. To evaluate model fit, we relied on the following recommendations (Hu & Bentler, 1999): Comparative fit index (CFI, values of > 0.90), and the Tucker-Lewis index (TLI, values of > 0.90); and the Root mean square error of approximation (RMSEA, values of < 0.06). To test if growth was predicted by sex and pubertal status at age 14, the intercepts and slopes of sending and receiving sexts, respectively, were regressed on these variables. Because puberty is closely linked to age, the exact age at baseline (using date of birth and exact assessment date at baseline) was included as a covariate.

## Results

### Prevalence and Characteristics of Sexting Behavior

Lifetime prevalence of sexting behavior is displayed in Table 2. The results show that at age 14, less than 8% of participants had sent sexts, whereas nearly 40% had received sexts at the same age. At age 18, the corresponding numbers for sending and receiving were 39% and 74%, respectively. Participants most typically sent sexts to boyfriends/girlfriends. At all timepoints, participants most frequently received sexts from unknown people. At age 18, approximately 32% reported to have received sexts from boyfriends/girlfriends, the corresponding number for age 16 being 23%, and 4% at age 14. Adolescents usually used their own social media platforms (non-anonymous) when sending sexts, and they most typically received sexts on their own platforms.

**Table 2:** Prevalence and characteristics of sexting behavior

	Age 14			Age 16			Age 18		
	Combined (n = 635) (%)	Boys (n = 299) (%)	Girls (n = 336) (%)	Combined (n = 664) (%)	Boys (n = 299) (%)	Girls (n = 365) (%)	Combined (n = 633) (%)	Boys (n = 288) (%)	Girls (n = 345) (%)
Sent sexts ever? %									
Yes	7.8	6.5	8.8	31.3	26.7	34.7	38.7	37.0	40.0
No	92.2	93.2	91.2	68.7	73.3	65.3	61.3	63.0	60.0
If yes—how many times?									
1 time	43.2	30.0	55.2	9.4	7.5	10.5	8.8	7.6	9.6
2–3 times	31.6	40.3	23.7	39.6	41	38.8	32.6	33.8	31.9
4–6 times	11.3	10.6	12.0	20.2	20.3	20.1	14.9	11.2	17.3
7 times or more	13.9	19.0	9.2	30.8	31.1	30.6	43.7	47.4	41.2
To whom have you sent?									
Unknown people	0.9	0.6	1.1	3.1	3.0	3.2	2.7	2.5	2.9
Friends	4.2	4.1	4.3	12.8	11.3	13.9	12.3	13.8	11.2
Boyfriend/girlfriend	3.4	4.4	2.6	19.0	14.5	22.5	27.3	22.7	30.7
Others	1.4	0.7	2.0	4.1	3.5	4.5	7.0	6.9	7.1
Sent where?									
Anonymous platforms	0.2	0.2	0.2	1.1	0.8	1.3	1.5	1.7	1.4
Social media platforms	6.4	5.1	7.5	24.0	20.2	26.9	31.6	27.7	34.6
Other places	1.6	2.3	1. 0	4.3	4.3	4.4	5.5	6.8	4.5
Received sexts ever?									
Yes	39.2	32.9	44.6	69.9	63.5	74.7	73.9	67.7	78.6
No	60.8	67.1	55.4	30.1	36.5	25.3	26.1	32.3	21.4
If yes—how many times?									
1 time	25.1	22.8	26.6	8.5	3.8	11.4	7.5	3.6	10.0
2–3 times	33.7	30.9	35.6	27.9	28.6	27.5	24.8	22.5	26.2
4–6 times	19.2	17.3	20.4	18.3	15.2	20.3	18.2	13.3	21.3
7 times or more	22.0	29.1	17.3	45.3	52.4	40.8	49.6	60.5	42.5
Received from whom?									
Unknown people	19.4	10.9	26.8	35.7	18.3	49.1	39.8	21.2	53.7
Friends	12.4	17.8	7.8	24.0	30.3	19.1	23.6	30.9	18.2
Boyfriend/girlfriend	4.3	6.2	2.6	23.0	25.7	21.0	32.3	36.3	29.3
Others	8.1	5.8	10.0	14.9	17.6	12.7	15.3	15.8	14.9
Received where?									
Anonymous platforms	2.6	1.4	3.6	3.1	2.3	3.8	4.8	4.2	5.2
Social media platforms	23.3	19.6	26.5	48.0	42.3	52.4	58.2	49.1	65.0
Other places		10.2	9.9	13.1	13.7	12.7	11.9	15.1	9.5

### Age Differences in Prevalence and Characteristics of Sexting Behavior

The prevalence of participants who had sent sexts increased with age (i.e., significant deterioration of model fit if thresholds are constrained to be equal across age) (Table 3). Regarding whom they had sent to, there was an increase in the proportion of all categories of receivers, except that participants were no more likely to send sexts to unknown people and friends at age 18 compared to age 16. Furthermore, as shown in Table 2, the percentage of participants who reported having used the three platforms examined (i.e., anonymous platforms, own social media platforms, and other places) increased with age. This age difference was indeed significant: From age 14 to 16 and 16 to 18, respectively, there was an increase in the number of youth reporting to have sent sexts using their social media site (Table 3). There was also a significant increase in the number of youth reporting to use anonymous platforms or “other places”, but only from age 14 to 16 and 18, respectively (i.e., not 16–18 years).

**Table 3:** Age differences in prevalence and characteristics of sexting

Variables	Age 14 vs 16			Age 16 vs 18			Age 14 vs 18		
	Δdf	Δχ^2^	*p*	Δdf	Δχ^2^	*p*	Δdf	Δχ^2^	*p*
Sent sexts									
Sent ever	1	111.20	< .001	1	13.59	< .001	1	148.14	< .001
To unknown	1	8.00	.005	1	0.31	.580	1	5.76	.016
To friends	1	33.55	< .001	1	0.10	.748	1	27.85	< .001
To boyfriend/girlfriend	1	72.27	< .001	1	19.80	.000	1	117.17	< .001
To others	1	6.29	.012	1	8.97	.003	1	22.13	< .001
On anonymous platforms	1	9.56	.002	1	0.24	.623	1	13.80	< .001
On social media platforms	1	83.62	< .001	1	16.97	< .001	1	129.65	< .001
On other places	1	7.63	.006	1	0.76	.383	1	12.46	< .001
Received sexts									
Received ever	1	122.53	< .001	1	3.04	.081	1	140.41	< .001
From unknown	1	46.21	< .001	1	2.59	.108	1	60.10	< .001
From friends	1	31.85	< .001	1	0.03	.859	1	29.17	< .001
From boyfriend/girlfriend	1	91.08	< .001	1	20.41	< .001	1	141.51	< .001
From others	1	15.45	< .001	1	0.09	.759	1	17.66	< .001
On anonymous platforms	1	0.61	.435	1	1.43	.232	1	3.57	.059
On social media platforms	1	88.37	< .001	1	18.16	< .001	1	142.60	< .001
On other places	1	2.49	.115	1	.44	.607	1	1.00	.317

*Note* Satorra-Bentler scaled chi-square tests were used

Age increases in receiving sexts were most prominent from 14 to 16 years. The prevalence of those who reported to have received sexts did not significantly increase from age 16 to 18 years, and neither did the proportion of those having received sexts from unknown people, friends, and others. However, the number reporting to have received sexts from a boyfriend/girlfriend rose significantly from each time point to the next. The same steady increase was found for the proportion of participants who reported having received sexts on their social media platforms. Participants were not more likely to receive sexts on anonymous platforms or “other places” as they got older.

### Sex Differences in Prevalence and Characteristics of Sexting Behavior

We found no sex differences in the proportion of participants who reported having sent sexts, at any time point (Table 4). Only one sex difference was revealed for characteristics of sending: at age 16, girls were more likely than boys to have sent sexts to boyfriends/girlfriends.

**Table 4:** Sex-differences in prevalence and characteristics of sexting

Variables	Age 14			Age 16			Age 18		
Sent sexts	B (95% CI)	*p*	OR (95% CI)	B (95% CI)	*p*	OR (95% CI)	B (95% CI)	*p*	OR (95% CI)
Sent ever	− .29 (− .96, .38)	.397	.75 (.38, 1.46)	− .38 (− .76, .01)	.056	.69 (.47, 1.01)	− .08 (− .45, .30)	.682	.93 (.64, 1.35)
To unknown	.76 (− 1.07, 2.60)	.415	2.15 (34, 13.48)	− .04 (− 1.04, .96)	.943	.96 (.36, 2.62)	.06 (− 1.00. 1.12)	.911	1.06 (.37, 3.08)
To friends	.06 (− .079, .91)	.891	1.06 (.45, 2.49)	.22 (− .31, .74)	.421	1.24 (.73, 2.10)	− .29 (− .83, .26)	.298	.75 (.44, 1.29)
To boyfriend/girlfriend	− .53 (− 1.50, 44)	.281	.59 (0.22, 1.55)	.54 (.09, 1.00)	.019	1.72 (1.09, 2.71)	.37 (− .03, .78)	.070	1.45 (.97, 2.18)
To others	1.03 (− .71, 2.77)	.246	2.80 (.49, 15.90)	.22 (− .69, 1–13)	.638	1.25 (.50, 3.11)	.03 (− .68, .75)	.927	1.03 (.51, 2.11)
On anonymous platforms	.71 (− 2.07, 3.49)	.616	2.03 (.13, 32.69)	.42 (-1.32, 2.16)	.639	1.52 (.27, 8.63)	− .23 (− 1.66, 1.21)	.759	.80 (.19, 3.36)
On social media platforms	.42 (− .29, 1.13)	.248	1.52 (.75, 3.10)	.34 (− .07, .75)	.103	1.41 (.93, 2.12)	.25 (− .13, .64)	.200	1.29 (.88, 1.89)
On other places	− .92 (− 2.38, .53)	.214	.40 (.09, 1.70)	.06 (− .80, .91)	.898	1.06 (.45, 2.49)	− .49 (− 1.26, .29)	.222	.62 (.28, 1.34)

Variables	Age 14			Age 16			Age 18		
Received sexts	B (95% CI)	*p*	OR (95% CI)	B (95% CI)	*p*	OR (95% CI)	B (95% CI)	*p*	OR (95% CI)
Received ever	− .56 (− .93, − .18)	.004	.57 (.93, .84)	− .56 (− .94, − .17)	.005	.57 (.39, .84)	− .54 (− .95, − .14)	.009	.58 (.39, .87)
From unknown	1.16 (.68, 1.65)	< .001	3.19 (1.97, 5.18)	1.46 (1.06, 1.86)	< .001	4.31 (2.88, 6.44)	1.46 (1.05, 1.86)	< .001	4.29 (2.86, 6.42)
From friends	− .92 (− 1.46, − .38)	< .001	.40 (.23, .69)	− .64 (− 1.04, − .24)	.002	.53 (.35, .79)	− .72 (− 1.14, − .29)	< .001	.49 (.32, .75)
From boyfriend/girlfriend	− .89 (− 1.80, .03)	.057	.41 (.17, 1.039	− .24 (− .64, .17)	.255	.79 (.16, 1.19)	− .33 (− .71, .05)	.087	.72 (.49, 1.05)
From others	.61 (− .06, 1.27)	.072	1.83 (.95, 3.55)	− .46 (− .95, .02)	.061	.63 (.39, 1.02)	− .09 (− .59, .41)	.729	.92 (.56, 1.51)
On anonymous platforms	1.14 (.02, 2.27)	.047	3.13 (1.02, 9.65)	.58 (− .47, 1.64)	.277	1.79 (.63, 5.14)	.20 (− .65, 1.06)	.644	1.22 (.52, 2.87)
On social media platforms	.43 (.01, .84)	.043	1.53 (1.01, 2.32)	.38 (.03, .72)	.032	1.46 (1.03, 2.06)	.60 (.24, .96)	< .001	1.82 (1.27, 2.61)
On other places	− .02 (− .59, 56)	.959	.99 (.56, 1.75)	− .10 (− .61, .41)	.607	.90 (.54, 1.50)	− .55 (− 1.09, .99)	.052	.58 (.34, 1.004)

Regarding receiving, several sex differences were found: At all ages, more girls than boys reported having received sexts. At all ages, girls were also more prone to receive sexts from unknown people compared to boys, whereas boys were more likely than girls to receive sexts from friends. Significantly more girls than boys reported having received sexts on their social media platforms at ages 14, 16, and 18 years, and having received sexts on anonymous platforms at age 14.

Bivariate correlations between sending and receiving are displayed in Table 5, showing moderate to strong cross-sectional associations between these sexting behaviors at all time points. The correlation between sending and receiving was significantly higher at ages 16 and 18 compared to age 14 (age 14 vs. 16: Δdf = 1; Δχ^2^ = 57.97; *p* ≤ .001; age 14 vs 18: Δdf = 1; Δχ^2^ = 109.90; *p* ≤ .001), thus youth being more likely to display both behaviors by increasing age. For both sending and receiving, there was significant two-year rank-order stability (i.e., stability relative to the group). From age 14 to 16, the stability was moderate, whereas strong associations (Cohen, 1992) were found from age 16 to 18 for both sexting behaviors.

**Table 5:** Bivariate correlations between frequency of sending and receiving at all time points (n = 743)

	Girls						Boys					
	1	2	3	4	5	6	1	2	3	4	5	6
1. Sending age 14	–	.19***	.14**	.43***	.17***	.08	–	.50***	.26**	.34***	.26***	.15*
2. Sending age 16		–	.63***	.14*	.61***	.42***		–	.62***	.22**	.58***	.43***
3. Sending age 18			–	.06	.46***	.59***			–	.13	.46***	.64***
4. Receiving age 14				–	.31***	.19***				–	.34***	.22***
5. Receiving age 16					–	.55***					–	.55***
6. Receiving age 18						–						–

*Note* *p < .05; **p < .01; ***p < .001

### Course of Sexting

Because the growth in sexting was not assumed to necessarily be linear, the growth in frequency of sending and receiving nudes at ages 14 and 18 was fixed to 0 and 4, respectively, whereas the age 16 value was freely estimated. For each of the two variables, the intercept was allowed to correlate with the slope. Further, the two intercepts were allowed to correlate with one another, as were the slopes. The fit of this model was not satisfactory (CFI = .891; TLI = .672; RMSEA = .155 (90% CI: .13, .18)). Based on the modification indices, cross-sectional associations between sending and receiving at ages 16 and 18 were added, suggesting that the covariation between sending and receiving at these ages (when sexting became prevalent) was not fully captured by the overall correlation between the intercepts and the slopes for the whole period. This yielded a good model fit (CFI = .999; TLI = .995; RMSEA = .019 (90% CI: .00, .07)).

The results showed that the growth of frequency in lifetime sending and receiving per year was 1.54 and 1.22, respectively (i.e., means of the slopes), with recoded variables applied (0 = 0; 1 = 1; 2 = 2.5; 3 = 5; 7 = 10). Thus, in actual frequency numbers, the increase in lifetime frequency was between 1 and 2.5 per year across ages 14 to 18. The means of the intercepts were 1.11 for sending and 1.66 for receiving, respectively. The two intercepts were highly correlated (*r* = .49, 95% CI = .22, .75,* p* ≤ .001), as were the two slopes (*r* = .68, 95% CI = .44, .92, *p* ≤ .001).

In a second step, sex, exact age at assessment (using date of birth and assessment date), and pubertal status at baseline (i.e., the age 14 assessment) were included as predictors. This model also yielded good fit (CFI = .987; TLI = .962; SRMR = .032; RMSEA = .038 (90% CI: .02, .06). As displayed in Table 6, sex, pubertal status, and age did not predict slopes of sending and receiving. Regarding the intercepts though, those 14-year-olds born early in the year (and thus being older) had higher intercepts of sending and receiving than those born later in the year, and those more pubertally advanced for their age also had a higher intercept of sending.

**Table 6:** Sex, age and pubertal status as predictors of intercept and growth in sending and receiving sexts (n = 743)

	Intercept				Growth			
Predictors	Sending		Receiving		Sending		Receiving	
	*β* (95% CI)	*p*	*β* (95% CI)	*p*	*β* (95% CI)	*p*	*β* (95% CI)	*p*
Sex	− .036 (− .13, .06)	.440	− .071 (− .03, .17)	.156	.060 (− .05, .17)	.302	.004 (− .11, .12)	.945
Puberty	.022 (− .06, .11)	.614	.135 (.03, .24)	.014	.031 (− .10, .16)	.630	− .032 (− ,16, .09)	*.*611
Age	.137 (.02, .26)	.027	.139 (.04, .24)	.008	− .027 (− .13, .08)	.620	− .097 (− . 21, .01)	.086

## Discussion

Using three waves of data from a birth cohort of Norwegian adolescents, we examined the prevalence, growth and characteristics of sexting behavior, identifying to whom adolescents sent sexts, from whom they received them, and to what degree they used anonymous or non-anonymous platforms when sexting, at ages 14, 16, and 18 years.

### Prevalence and Characteristics of Sexting

Our results showed that across ages, receiving sexts was much more common than sending sexts. At age 14, only 8% reported having sent sexts, the corresponding numbers at age 16 and 18 being 31% and 39%, respectively. A meta-analysis (n = 28) capturing ages 11–21 (mean age 14.9) found a prevalence of 19% for sending, and the prevalence increased with age (Mori et al., 2022). We further found that at age 14, 39% reported having received sexts, which corresponds to Mori et al.’s pooled prevalence rate of 35% (n = 14). Notably, the authors did not find age to moderate this estimate, whereas we found a significant increase in prevalence by age, with 74% reporting to have received sexts by age 18. Such an increase was also reported in an earlier meta-analysis (Madigan et al., 2018).

The finding that youth more frequently sent and received sexts as they aged is unsurprising, given the increasing interest in sex that comes with puberty. Adolescents’ sexual urges and activities become more directed toward interest in potential sexual partners, as opposed to the more diffuse and self-oriented pleasure-seeking from early childhood (Cacciatore et al., 2019). The initiation of partnered sexual activity is one of the main developmental tasks of adolescence. In such a context, it is also unsurprising that youth most typically sent sexts to boyfriends/girlfriends, with a significant increase from 19 to 27% across ages 16 to 18. The number was much lower at age 14 (3.4%), which is likely in part due to the lower rates of romantic relationships and sexual partnerships in early-to-middle adolescence, as compared to late adolescence (Beckmeyer et al., 2020). The number of participants who reported having received sexts from a boyfriend/girlfriend also significantly rose from each timepoint to the next, and at age 16 and 18, it exceeded the number reporting having received sexts from friends, which at age 14 was three times more frequently reported than receiving sexts from boyfriends/girlfriends. A study analyzing the content of text messages found that sexual communication more typically appeared in relation to romantic partners than peers across grades 9–12 (Ehrenreich et al., 2020). Others have also found that youth most typically send and receive sexts with romantic partners (Foody et al., 2021; Patchin & Hinduja, 2019). In sum, these findings accord with the view that for some youth, sending and receiving sexts is part of normative sexual development (Bianchi et al., 2017; Lebedíková et al., 2024; Mori et al., 2022; Temple et al., 2019; Thorne et al., 2024; Widman et al., 2021). It is also worth noting that the two-year rank-order stability in sexting was moderate from age 14 to 16, but strong from age 16 to 18. Thus, as age and sexual maturity increased, participants were more likely to display the same behavior across time (i.e., relative to the group). The correlation between sending and receiving was also significantly higher at ages 16 and 18 compared to age 14, which indicates that the two behaviors more typically go together in late adolescence compared to early-to-middle adolescence.

At age 14, 19% of our participants reported having received sexts from unknown people, a number that doubled by the age of 18. Although we cannot know whether these sexts were experienced as intrusive, and we lack information about whether sexts were nonconsensual, former research has shown that youth, and especially girls, are often exposed to online sexual harassment from unknown people (Jones et al., 2012). It is reasonable to assume that receiving sexts from unknown people feels more intrusive at younger compared to older ages; thus, our finding that one in five 14-year-olds reported this is concerning. It is also worth noting that among the categories of receivers assessed in the present inquiry, “unknown people” was the most prevalent at all ages. Future research should aim to explore whether receiving sexts from unknown people versus other categories of senders differently affects adolescents, and if so, what measures can be taken to protect against receiving such sexts. On a related note, we found that at all ages, less than 5% reported sending and receiving sexts on anonymous platforms. Sexting behavior most typically took place on the adolescents’ non-anonymous social media platforms (e.g., Snapchat, Instagram). Given the overall high number of adolescents who reported having received sexts from people they did not know, it is possible that some of these encounters with unknown people occurred through these traditional social media platforms. An important line of future inquiry concerns experiences of receiving sexts from people who are not personally known to the adolescent offline, but who are not operating anonymously.

### Sex Differences in Sexting

The overall prevalence of sending sexts did not significantly differ between girls and boys, which accords with existing research (Barrense-Dias et al., 2022; Foody et al., 2021; Mori et al., 2022). At age 16 though, girls were significantly more likely than boys to have sent sexts to their boyfriends/girlfriends, which aligns with prior research showing that girls are more frequently asked and more often feel pressured to send sexts (Burén & Lunde, 2018; Foody et al., 2021). Patchin & Hinduja (2019) on the other hand, found girls to be *less* likely than boys to have sent sexts to a romantic partner (age 12–17, mean age 14.5 years) and others have reported that *boys* feel more pressured than girls to sext with their romantic partners (Van Ouytsel et al., 2019).

In accordance with meta-analytical evidence (Mori et al., 2022), girls were found to be more likely than boys to receive sexts. We extend this evidence by showing that girls more often receive sexts from unknown people and on their social media platforms, which suggests they are more exposed to intrusive and unwanted sexts than boys. A Swedish study also reported girls to be more likely to receive sexts from strangers (Burén & Lunde, 2018). Thus, even in gender-equal and sexually liberal countries such as Norway and Sweden, girls tend to receive unwanted sexts and feel pressured to send them more often than boys (Burén & Lunde, 2018), making them more vulnerable to sexualization. Interestingly, cross-cultural studies of sex differences in other sexually related behaviors—such as regret after casual sex or sexual jealousy—show similar patterns in Norway as in less egalitarian and sexually liberal countries (Bendixen et al., 2015; Kennair et al., 2016). In sum, these findings do not support the biosocial role theory (Eagly & Wood, 1999).

### The Course of Sexting

Our growth analyses indicated that the increase in actual frequency of lifetime sending and receiving was rather small, with a yearly increase of 1–2.5 sexts. Please note that the highest response category was 7 times or more, but even at age 18, less than 50% of those stating that they have sent sexts indicated that they had done so 7 times or more. Among U.S. adolescents who reported having sent sexts—defined as having sent images—one third reported having done so only once (Patchin & Hinduja, 2019), and a Swiss study of middle-school adolescents found that among those who reported having sent sexually related images, most had done it only once (Barrense-Dias et al., 2022). Finally, Steinberg et al. (2019) found that 50% of 11th graders had at least one incidence of sexting (sending sexual messages or pictures), the corresponding number at grades 10 and 9 being 37% and 24%, respectively, thus quite similar prevalences as found in our sample. In sum, the above numbers suggest that the frequency range captured, as well as the prevalence and yearly increase identified in the present inquiry, is reasonably well-aligned with prior work. As noted by Patchin and Hinduja (2019), defining sexting as sending/receiving sexually related images or nudes is substantially different than definitions that also include text-based messages, given the different social and legal consequences that may come with these differing sexting contents. Studies examining text messages indeed tend to report much higher numbers than the ones displayed above (e.g., Kurup et al., 2022). As noted above though, in the Mori et al. (2022) meta-analysis, sexting content did not moderate the prevalence estimates. However, the authors compared studies assessing sending/receiving images only with those capturing mixed content, also including videos—and examined prevalence, not frequency—thus not being positioned to test potential differences in how often youth send/receive textual messages vs. images and videos.

Finally, aiming to differentiate between the role of age, sex and pubertal status in the development of sexting—and thus account for their interrelated nature—we tested if these predicted the starting point (i.e., intercept) and course (i.e., slope/growth) of sexting. We found that for both sexting behaviors, the intercept or starting point of the growth was positively predicted by age (i.e., those who were relatively older at the age 14 assessment reported more sexting), and the intercept of sending was positively predicted by pubertal status (i.e., those who were more pubertally mature reported more sending behavior). This accords with meta-analytical evidence showing that more pubertally advanced adolescents are more likely to engage in sexual behavior and risky sexual behavior (Baams et al., 2015), with some survey studies also directly linking early pubertal status to increased likelihood of sexting (Houck et al., 2014), at least when it comes to sexting with romantic partners (Burén & Lunde, 2018). In the present inquiry, however, none of the predictors affected growth in frequency of sending and receiving sexts from age 14 to 18. Thus, although those who are “early bloomers” may be prone to start sending sexts earlier than those who are less physically mature, in our study, pubertal timing did not affect future changes (i.e., increases or declines) in frequency of sexting. Neither pubertal status, nor sex or biological age, had an “accelerating effect” on sexting. Future studies should examine other potential predictors, such as having a romantic partner.

### Limitations

The strengths of the study include the use of a large birth-cohort sample assessed at three timepoints over 6 years, capturing detailed information about adolescents’ sexting. However, it should be acknowledged that we assessed the frequency of lifetime sexting (i.e., if you have ever sent/received sexts, how often…), which may be less reliable than assessing the current frequency of sexting (e.g., in the last month…) as it puts more demands on the participants’ recall abilities. On the other hand, the specificity of the questionnaire items may enhance recall and promote accurate responses compared to broader terms or labels (i.e., sexting). Further, the study did not include information on whether the sexts were consensual or not, which will be a critical direction for future research. Similar to the majority of sexting research, we assessed self-reported sexting behavior, which may be less valid than objective measures, such as analyzing the content of participants’ digital activity. However, the latter comes with ethical considerations and logistical challenges, particularly in a large and prospective study. Finally, a substantial challenge in research on digital media in general, and prospective studies such as this one in particular, is the fast-developing technology (e.g., new apps and platforms), which may affect online behavior such as sexting. Also, although meta-analytical evidence suggests that study location (i.e., North America vs Europe) does not moderate the prevalence estimates of sexting, some studies do find the frequency of sexting to differ between countries (e.g., Mexico and Colombia vs Spain), although other characteristics do not (e.g., age, sex) (Gil-Llario et al., 2020, 2021). Nevertheless, the present findings may not generalize to cultures that are substantially different from Western societies.

### Conclusion

Using three waves of biennially collected data from a Norwegian birth cohort of adolescents, the present study found that across ages 14 to 18, receiving sexts was much more common than sending sexts. Girls were no more likely than boys to send sexts but were more likely to receive them. Both the prevalence of sending and receiving sexts increased by age. At ages 16 and 18, the receiver was most typically a romantic partner. Even so, across age, participants most typically received sexts from unknown people, with one in five reporting this at age 14. In sum, our findings underscore that interventions and policies should acknowledge that certain types of sexting can be a developmentally appropriate aspect of sexual development, while at the same time developing measures to protect against sexual victimization and risks associated with sexting.

## Supplementary Information

Below is the link to the electronic supplementary material.

Supplementary file1 (DOCX 65 KB)

## Data Availability

The data cannot be made available because the study is still ongoing, and participant consent restrictions apply.

## Code Availability

The code is made available by request from the corresponding author.
