# Correction: Development and Characteristics of Sexting from Age 14 to 18 Years in a Norwegian Birth Cohort

**DOI:** 10.1007/s10508-026-03486-2

**Published:** 2026-04-16

**Authors:** Silje Steinsbekk, Jacqueline Nesi, Sophia Choukas-Bradley, Lars Wichstrøm

**Affiliations:** 1 Department of Psychology, Norwegian University of Science and Technology, Dragvoll, 7491 Trondheim, Norway; 2 Department of Psychiatry & Human Behavior, Warren Alpert Medical School of Brown University, Providence, RI USA; 3 Department of Psychology, University of Pittsburgh, Pittsburgh, PA USA; 4 Department of Child and Adolescent Psychiatry, St. Olavs University Hospital, Trondheim, Norway

**Correction to: Archives of Sexual Behavior** 10.1007/s10508-026-03413-5

The first sentence in the **Measures** section was incorrect in the article as originally published and should have read:

At all three measurement points (ages 14, 16, and 18), participants responded to 8 items capturing whether they had ever (1) sent to others or published online an image or video of themselves wearing little clothing (e.g., with breasts or genitals partly visible), and (2) received an image or video of another person who was wearing little clothing. The items also assessed to whom such content had been sent, from whom it had been received, and which platforms were used (e.g., their own social media accounts, anonymous platforms, or other platforms).

In addition, in Table 3 the variable category “Sent sexts” was misplaced as a table header instead of appearing under the table header “Variables”.

The original article has been updated to incorporate all needed corrections.

