# Supplementary material for: Development and Characteristics of Sexting from Age 14 to 18 Years in a Norwegian Birth Cohort
Source: Arch Sex Behav. 2026 Mar 17;55(3):1345–57. doi: 10.1007/s10508-026-03413-5 (PMC13194284; doi:10.1007/s10508-026-03413-5)
Supplement: Supplementary file 1 — Supplementary file1 (DOCX 65 KB) [file 10508_2026_3413_MOESM1_ESM.docx]

**Figure. S1**. The procedure and flow of participants


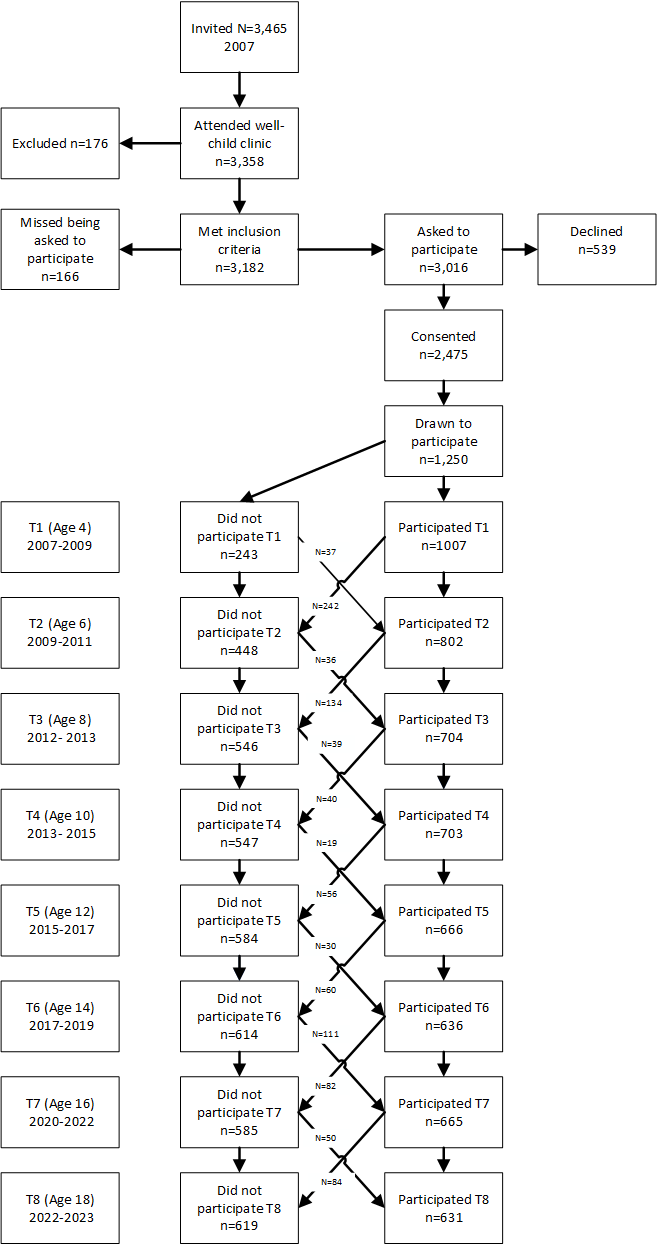


*Note:* The number of participants at the various assessment points is based on the number of participants invited to participate, subtracted from those who did not participate at the respective measurement points.
